# Data-based Reconstruction of Gene Regulatory Networks of Fungal Pathogens

**DOI:** 10.3389/fmicb.2016.00570

**Published:** 2016-04-22

**Authors:** Reinhard Guthke, Silvia Gerber, Theresia Conrad, Sebastian Vlaic, Saliha Durmuş, Tunahan Çakır, F. E. Sevilgen, Ekaterina Shelest, Jörg Linde

**Affiliations:** ^1^Research Group Systems Biology and Bioinformatics, Leibniz Institute for Natural Product Research and Infection Biology – Hans Knoell InstituteJena, Germany; ^2^Computational Systems Biology Group, Department of Bioengineering, Gebze Technical UniversityKocaeli, Turkey; ^3^Department of Computer Engineering, Gebze Technical UniversityKocaeli, Turkey

**Keywords:** *Candida albicans*, *Aspergillus fumigatus*, reverse engineering, text mining, transcription factor, genome-wide modeling

## Abstract

In the emerging field of systems biology of fungal infection, one of the central roles belongs to the modeling of gene regulatory networks (GRNs). Utilizing omics-data, GRNs can be predicted by mathematical modeling. Here, we review current advances of data-based reconstruction of both small-scale and large-scale GRNs for human pathogenic fungi. The advantage of large-scale genome-wide modeling is the possibility to predict central (hub) genes and thereby indicate potential biomarkers and drug targets. In contrast, small-scale GRN models provide hypotheses on the mode of gene regulatory interactions, which have to be validated experimentally. Due to the lack of sufficient quantity and quality of both experimental data and prior knowledge about regulator–target gene relations, the genome-wide modeling still remains problematic for fungal pathogens. While a first genome-wide GRN model has already been published for *Candida albicans*, the feasibility of such modeling for *Aspergillus fumigatus* is evaluated in the present article. Based on this evaluation, opinions are drawn on future directions of GRN modeling of fungal pathogens. The crucial point of genome-wide GRN modeling is the experimental evidence, both used for inferring the networks (omics ‘first-hand’ data as well as literature data used as prior knowledge) and for validation and evaluation of the inferred network models.

## Introduction

While most of fungal species are harmless for human, some can cause infections (mucoses) with very high mortality rates. The number of fungal infections has increased over the last decades due to the rising number of immunocompromised patients (Pfaller and Diekema, 2010). Mucoses are associated with excessive morbidity and mortality because of difficulties with diagnosis and antifungal therapy. Fungal pathogens have evolved diverse strategies for colonisation, adhesion, invasion, translocation, nutrient acquisition, interaction with immune cells of the host, and (secondary) metabolite synthesis (Brakhage, 2013). About 600 fungal species have been reported to infect human (Brown et al., 2012a). Over 90% of fungal infections resulting in death are caused by *Candida*, *Cryptococcus*, *Aspergillus*, and *Pneumocystis.* These species cause over two million life-threatening infections each year (Brown et al., 2012b). Species belonging to the genera *Candida* and *Aspergillus* are widely recognized as the most important human fungal pathogens (Moran et al., 2011).

A key feature of fungal infections is the interaction between different morphotypes of fungal cells (colonizing, persisting, or invading) and all kinds of tissues and immune effector cells of the host. Because of their complexity, the elucidation of invasive fungal infections requires a systems biological approach for modelling the complex interactions between a large number of genes, proteins, metabolites, cells, and tissues from both the pathogen(s) and the host (Forst, 2006). For reviews of the systems biology of infections caused by the human pathogenic fungi *Candida albicans* and *Aspergillus fumigatus*, (see e.g., Albrecht et al., 2008, 2011; Rizzetto and Cavalieri, 2011; Horn et al., 2012, 2014).

The dynamics of host-pathogen interactions has to be analyzed and modeled in temporal and spatial resolution on different scales. Recently, we reviewed the emerging multi-scale modeling of bacterial and microbial infections (Schleicher et al., 2016). Dühring et al. (2015) reviewed interaction mechanisms between the human innate immune system and *C. albicans* focussing on the mathematical modeling of immune evasion strategies. These molecular interactions can be modelled by networks composed of nodes (molecules) connected by edges (interactions). Network models of host–pathogen interactions, such as protein–protein interactions, signaling, metabolic, and gene regulatory networks (GRNs) can be computationally reconstructed by exploiting molecular databases, literature mining and model fit to experimental data (Durmuş et al., 2015).

Focussing on the transcriptomic scale, here we review fungal GRN models, where nodes represent genes and edges describe causal regulator–target gene relations. GRN reconstruction is mainly based on transcriptome data supported by prior knowledge interactions derived from literature. Additionally, a set of experimentally verified interactions is needed as ‘gold standard’ for validation of the inferred model (Hecker et al., 2009; Linde et al., 2015). In the present article, we compare the feasibility of genome-wide vs. small-scale modelling under the current state of (i) species-specific interactome knowledge, (ii) prior knowledge retrieved via orthologous genes in other fungal species, (iii) transcriptome data for model fit, and (iv) confirmed knowledge for model validation.

Here, we discuss GRN prediction for *A. fumigatus* – one of the most important causes of life-threatening invasive mycoses. While a genome-wide network model for for *C. albicans* has been published based on a compendium of microarray data and knowledge extracted by molecular database search and literature mining (Altwasser et al., 2012), we here show that genome-wide GRN modeling with sufficient quality is currently not feasible for *A. fumigatus*. We found that this is due to a larger genome compared with *C. albicans* in conjunction with a lower number of known interactions and publicly available transcriptome data sets. Finally, systems biology of fungal infection requires modeling GRNs not only in the pathogen but also in the host and modeling of molecular interactions between them. This was demonstrated so far only for *C. albicans* inferring a small-scale model (Tierney et al., 2012).

## Species-Specific Genomic Knowledge About the Fungal Pathogens

The current knowledge about molecular interactions for *C. albicans* and *A. fumigatus* is relatively moderate compared with the comprehensive knowledge about the model eukaryote *Saccharomyces cerevisiae* that is represented in more than 100,000 scientific publications. Moreover, the knowledge about *A. fumigatus* is scarcer than that for *C. albicans* as shown in **Table 1**. For *C. albicans* with 6,218 genes (ORFs) there are more than 33,000 journal papers whereas for *A. fumigatus*, despite a larger genome size, there are less than 10,000 articles referenced in PubMed.

**Table 1:** Survey of available data for the fungi *Candida albicans* and *Aspergillus fumigatus.*

	*C. albicans*		*A. fumigatus*	
**Prior knowledge from species-specific databases and literature**
# ORFs	6,218	Candida Genome Database [CGD], 2016	9,840	Aspergillus Genome Database [ASPGD], 2016
# ORFs verified	1,581 25%	Candida Genome Database [CGD], 2016	483 5%	Aspergillus Genome Database [ASPGD], 2016
# TFs predicted	241	Candida Genome Database [CGD], 2016	273	Aspergillus Genome Database [ASPGD], 2016
# TFs (validated)	43	TRANSFAC, 2016	0	TRANSFAC, 2016
# Articles	33,205	PubMed, 2016	9,424	PubMed, 2016
**Prior knowledge from other databases via orthologs**
# Interactions # Genes	249 226	TRANSFAC, 2012	47 64	TRANSFAC, 2015
# Interactions # Genes	6,674 2,290	MPact, 2012	1,171 1,229	MPact, 2015
# Interactions # Genes	2,689 1,502	Balaji et al., 2006	231 234	Balaji et al., 2006
# Interactions # Genes	6,333 2,288	BIND, 2012	43,852 3,465	STRING, 2015
# Interactions # Genes			2,470 1,122	BioGRID, 2015
# Interactions	11,523	Union of all four	47,230	Union of all five
**Experimental gene expression data sets (for model inference)**
# Samples	198 1,846	Ihmels et al., 2005 GEO, 2016	101 79	GEO, 2015 unpublished
**Knowledge extracted by text mining for model validation (‘gold standard’)**
# Regulators # Interactions # Target genes	372 4,625 1,484	Literature mining + CGD	31 136 104	Literature mining + manual curation
# Interactions # Genes	1,016 503	Altwasser et al., 2012	321 273	as above + AspGD


*ORFs for C. albicans SC5314 and A. fumigatus Af293; for referencing of sources CGD, AspGD, TRANSFAC, etc. see text. STRING database filtered by score: text mining evidence OR experiment evidence OR database evidence ≥ = 700 AND combined score ≥ = 700.*

Curated databases have been developed to provide improved computational access to the biologically important information. Basic genomic knowledge about *C. albicans* and *A. fumigatus* is provided by the Candida Genome Database (CGD; Skrzypek et al., 2016) and *Aspergillus* Genome Databas (AspDB; Cerqueira et al., 2014), respectively. Additionally, functional categorisation of fungal genes and proteins can be extracted for 298 fungal strains by the Web tool FungiFun2 (Priebe et al., 2014).

In general, manually curated databases have high quality. However, they cannot be up-to-date as it takes time before new discoveries are included (Baumgartner et al., 2007). Therefore, the data presented in databases, such as CGD, AspDB, and TRANSFAC (Wingender, 2008), does not fully represent the current state of rapidly growing knowledge. Obviously, this is the case for TRANSFAC that does not provide yet any information about TFs in *A. fumigatus* (**Table 1**). The model fungus *S. cerevisiae* has an estimated number of 140 to 250 TFs (Costanzo et al., 2014) and about 600 proteins may be involved in the transcriptional regulation of approximately 6,000 protein-coding genes (Cherry et al., 2012). The number of TFs varies according to the selection criteria. Some studies count proteins with predicted DNA-binding domains; others include only proteins shown to bind DNA directly; still others also include non-DNA binding subunits of TF complexes. The regulatory proteins can be subdivided into the basal machinery, co-regulators, chromatin-remodeling and modifying factors, as well as DNA-binding TFs. We estimate for *A. fumigatus* a number of about 600 TFs (unpublished results). Compared to these estimated high number of TFs, the number of experimentally verified TFs that are included in TRANSFAC is very small. However, the list of predicted (putative) TFs is increasing. For instance, based on the first complete genome sequence for *A. fumigatus* Af293, a set of 28 transcriptional regulators (22 TFs) were predicted (Nierman et al., 2005). The AspDB currently assigns 273 gene loci to a TF.

## Prior Knowledge From Other Databases Via Orthologous Genes

An approach to fill the gap of missing species-specific knowledge is to make use of known interactions in closely related species via the mapping of orthologs taking into account the problem that the regulation of a gene might be different between phylogenetically close fungal species. Linde et al. (2011) and Altwasser et al. (2012) mapped orthologous genes of several fungal species including *S. cerevisiae*, *A. nidulans, C. glabrata* to *C. albicans* and screened four databases for transcriptional interactions: (i) TRANSFAC, (ii) MPact (Güldener et al., 2006), (iii) the transcriptional regulatory network for yeast by Balaji et al. (2006), and (iv) BIND (Bader et al., 2003). In this article, we present primary results from genome-wide network inference for *A. fumigatus.* Therefore, we exploited three of these [TRANSFAC, MPact and Balaji et al. (2006)] and additionally used the STRING database (Szklarczyk et al., 2015) and BioGRID (Chatr-Aryamontri et al., 2015). The numbers of gene regulatory interactions and genes involved are shown in **Table 1**.

## Fungal Transcriptome Data

To identify and validate unknown putative gene regulatory interactions, a compendium of experimental gene expression data should be collected and analyzed. Ihmels et al. (2005) published a collection of microarray-based transcriptome data for 6,167 genes of *C. albicans* under 198 experimental conditions. A search for *C. albicans* in the Gene Expression Omnibus database (GEO; Barrett et al., 2013) resulted in transcriptome data sets composed of 1,846 samples at 2016/01/06. Among them, 1,467 samples were analyzed by microarrays and 379 samples were analyzed by high-throughput sequencing (HTS; 263 of them using Illumina HiSeq 2500).

For *A. fumigatus*, Nierman et al. (2005) published the first gene expression data set. At 2016/01/06 GEO contained transcriptome data from 101 samples analyzed by HTS of which we used 81 (GEO: GSE55743, GSE55663, GSE55943, and GSE30579). In addition, we considered unpublished HTS data of 79 samples from *A. fumigatus*. To avoid incompatibility problems between HTS and microarray data, we restricted the data analysis for *A. fumigatus* to the HTS data from the total of 160 samples. The HTS data was preprocessed as described by Schulze et al. (2015).

## Knowledge Extracted By Text Mining for Model Validation

To provisionally fill the gap of knowledge between the high number of expected and the low number of verified TFs and their target genes, articles downloaded from PubMed (abstracts) or PMC (full text) were computationally scanned using a software developed by us (Durmuş et al., 2015). The articles were preprocessed to obtain relevant sentences which were parsed afterward using natural language processing.

For *C. albicans* more than 20,000 articles were scanned for interactions comprising the following words (and its derivatives): bind, regulate, promote, suppress, in/activate, enhance, overexpress, attenuate, induce, block, inhibit, repress. A number of 4,625 unique interactions (including 1,388 interactions added from CGD) between 372 regulators and 1,484 target genes were found (**Table 1**). In a previous study using a text mining tool (JRex), we scanned about 9,000 articles on *C. albicans* and found 1,016 interactions between 509 genes (Altwasser et al., 2012).

In contrast, for *A. fumigatus* we scanned 1,580 open-access full-text articles and 6,420 abstracts of non-open-access articles. Automatic identification of 136 regulatory interactions between 28 regulators and 104 target genes complemented by manually curated interactions resulted in 31 regulators (including 15 TFs, **Supplementary Table S1**) and a total of 153 regulatory interactions. Based on the aforementioned knowledge for *A. fumigatus*, the well-known TFs SrbA, SreA, LaeA, BrlA, and Yap1 were identified as hubs (>7 regulator-target gene interactions). After addition of further interactions retrieved from AspGD, we obtained for *A. fumigatus* a set of 321 gene regulatory interactions between 273 genes, which was used as ‘gold standard’ for GRN model validation as described in the next section. This shows that despite the larger size of the genome, the numbers of known interactions are significantly smaller for *A. fumigatus* than for *C. albicans*.

## Large-Scale Modeling of GRN in Pathogenic Fungi

**FIGURE 1:**
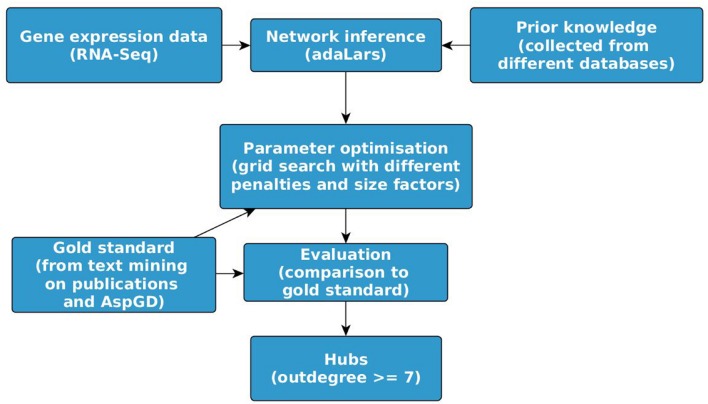
**Workflow for large-scale GRN inference.** For the example GRN for *Aspergillus fumigatus*, the network was inferred using the software package ‘adaLARS,’ an adaptive LASSO algorithm (Zou, 2006) combined with ridge regression as used by Altwasser et al. (2012). The prior knowledge was collected for this example from different databases as listed in **Table 1** under ‘Prior knowledge from other databases via orthologs.’ The Gold standard was extracted as described in section ‘Knowledge extracted by text mining for model validation.’ The hubs were identified using the tool igraph (Csardi and Nepusz, 2006).

There exists an impressive corpus of studies in the field of mathematical modeling and identification of GRN. For reviews see Hartemink (2005), Hecker et al. (2009), Huang et al. (2009), Goldenberg et al. (2010), and Linde et al. (2015). Genome-scale network models arose shortly after the first genome sequencing, starting with constraint-based metabolic network models (Bordbar et al., 2014). Most of the published genome-wide models are knowledge-based. Protein–protein interaction network models of *C. albicans* and *A. fumigatus* were published (Remmele et al., 2015) based on sequence similarity of known interaction and supported by GO enrichment analysis. As the available knowledge for the fungi, in particular for *A. fumigatus*, is scarce and far from complete, novel interactions have to be predicted by the integration of experimental data into the process of model construction. Due to the availability of data from only less than 200 samples compared to about 10.000 genes (see **Table 1**), low complex models were fitted, e.g., by regression of linear models. This was performed for *C. albicans* (Altwasser et al., 2012) using the workflow depicted in **Figure 1**, but to the best of our knowledge not yet for *A. fumigatus* and other human pathogenic fungi. Altwasser et al. (2012) inferred a linear regression model describing the interactions between regulators (expression intensity *x*_j_) and target genes (*x*_i_) of *N* (6,167) genes for different experimental conditions (samples *m*) with penalty defined by the prior knowledge:

$$x_{i}\,\left(m\right)=\overset{N}{\underset{j=1,j\neq i}{\Sigma }}\,\beta_{i,j}\,x_{j}\,\left(m\right)$$

The identification of the parameters β_i,j_ was performed using an adaptive LASSO (Least Absolute Shrinkage and Selection Operator) combined with ridge regression.

As the ‘gold standard’ for model validation in Altwasser et al. (2012) comprised only 503 genes, the refined GRN inference was restricted to this subset genes. The model was fitted to the transcription data collected by Ihmels et al. (2005) with 198 samples.

Here, we present preliminary results of genome-wide modeling for *A. fumigatus* based on HTS data from 160 samples and the knowledge (prior knowledge and ‘gold standard’) surveyed in **Table 1**. The workflow is depicted in **Figure 1** and the inferred network with highlighted hubs in the **Supplementary Figure S1**. The precision and sensitivity of the model quantified by the F-measure of inferred genome-wide GRN is low due to several reasons: the number of samples (160) is very small compared with the number of genes (almost 10,000). This is called the dimensionality problem (‘curse of dimensionality’). In general, this problem can be reduced by integration of prior knowledge. However, for both *C. albicans* and *A. fumigatus*, the overlap of the prior knowledge extracted from different databases and the ‘gold standard’ (obtained by text mining) is very small (as shown for *C. albicans* by Altwasser et al., 2012). For *A. fumigatus*, the overlap between the 47,230 interactions used as prior knowledge (including non-species-specific data) and the 321 interactions used as ‘gold standard’ (species-specific knowledge) is only 25 – indicating the high diversity of fungal species with respect to gene regulation. Nevertheless, despite the modest quality of the inferred large-scale network model for *A. fumigatus*, the identified hubs were found to be independent of the parameters of the inference algorithm.

## Small-Scale Modeling of GRN in Pathogenic Fungi

While the quality of genome-wide models for pathogenic fungi is low due to (i) the moderate amount of publicly available transcriptome data and (ii) the sparse quality and quantity of experimentally verified knowledge, there are some examples for the inference of small-scale modeling of GRNs for *C. albicans* and *A. fumigatus* with sufficient reliability. Using the NetGenerator algorithm (Guthke et al., 2005; Weber et al., 2013), a first model comprising four genes (Guthke et al., 2007) was presented for *A. fumigatus* based on transcriptome data published by Nierman et al. (2005). Later, small-scale networks for *A. fumigatus* with 12 and 26 genes were published by Linde et al. (2012) and Altwasser et al. (2015), respectively. Small-scale GRN models for *C. albicans* were also inferred using the NetGenerator algorithm and presented by Linde et al. (2010), Tierney et al. (2012), Ramachandra et al. (2014), Schulze et al. (2015), and Böhringer et al. (2016), whereas the articles on small-scale GRN inference used the top–down approach and simulated the expression profiles of selected genes using ordinary differential equations, Brandon et al. (2015) applied a bottom up approach to investigate the iron acquisition and oxidative stress response in *A. fumigatus* by discrete dynamic simulation of Boolean network models.

In all cases, the critical challenge in modeling of small-scale GRNs is the selection of genes. Mostly, the feature selection is supported by both prior knowledge and unsupervised learning algorithms, e.g., clustering or module identification.

An advantage and benefit of small-scale modeling is the opportunity to predict a small set of hypotheses about previously unknown gene regulatory interactions, that needs to be verified experimentally as done for the models by Linde et al. (2012), Tierney et al. (2012), Altwasser et al. (2015) and Böhringer et al. (2016). In these cases, small-scale GRN models supported the experimental design for the follow-up investigation of transcriptomic regulation in pathogenic fungi.

## Future Research

The molecular understanding of fungal pathogens will become more important as there are an ever-growing number of drug-resistant strains of human pathogenic fungi and an increasing number of immunocompromised individuals. Most certainly, the modeling of omics data will pave the way for predictive diagnostics and personalized treatment of fungal infections (Oliveira-Coelho et al., 2015). In future this modeling approach will be extended from the molecular to the cellular, organism, and population scale (Schleicher et al., 2016).

The modeling of GRNs is crucial to predict potential drug targets for improved treatment of fungal infections. One of the aims of GRN modeling is to identify ‘hubs,’ i.e., important transcriptional regulators, which are highly connected. Therefore, it is necessary to reconstruct the topology of the GRNs as precise as possible. Currently, only small-scale GRN models were inferred with sufficient reliability, i.e., their *in silico* predictions could be experimentally verified. The disadvantage of small-scale models is the need of feature selection, i.e., the selection of a number of genes of interest. Such investigation is hypothesis-driven and does make sense only in connection with a certain focussed scientific question. In contrast, systems biology claims a holistic perspective, which requires large-scale GRN modeling. We reviewed here the feasibility for large-scale GRN modeling for the two most important human pathogenic fungi *C. albicans* and *A. fumigatus.* For *C. albicans* such a model was previously published, but it could now be improved based on (i) the increased amount of transcriptional data, (ii) enhanced set of prior knowledge, and (iii) extended set of knowledge extracted by text mining for model validation as shown in **Table 1**.

In contrast, for *A. fumigatus* the quantity and quality of data and knowledge have to be improved before the time will be ripe for genome-wide GRN modeling. These requirements will be important for efficient genome-wide GRN modelling with high reliability also for other fungi: (i) curation of a more comprehensive database of TFs and TF-binding sites of human pathogenic fungi, (ii) more standardized reporting of results in the scientific literature using ontologies and controlled vocabulary, (iii) improved text mining algorithms to extract knowledge about gene regulatory interactions to reduce the effort for manual curation of the databases, (iv) increase in the number of gene expression data sets covering more experimental conditions of fungi in public repositories, (v) investigation on comparative genomics with respect to gene regulation pattern of different fungi to improve a more careful inclusion or exclusion of non-species-specific prior knowledge into the inference of large-scale GRN models. Currently, reliable genome-wide GRN modeling with some thousands of genes is impossible due to the scarce knowledge for model validation, i.e., there is no confirmed ‘gold standard.’ Today, the size of network models has to be adapted to the number of some dozens or hundreds of genes with sufficient knowledge about gene regulatory interactions.

## Author Contributions

RG and JL conceived and drafted the manuscript. SG collected and processed the transcriptome data for *Candida albicans* and *Aspergillus fumigatus* and performed the network inference for *A. fumigatus*. TC and SV contritubed to the network inference algorithms. SD, TC and FS performed the text mining. ES contributed to the transcription factors. All approved the final manuscript.

## Conflict of Interest Statement

The authors declare that the research was conducted in the absence of any commercial or financial relationships that could be construed as a potential conflict of interest.
