# Supplementary material for: Data-based Reconstruction of Gene Regulatory Networks of Fungal Pathogens
Source: Front Microbiol. 2016 Apr 22;7:570. doi: 10.3389/fmicb.2016.00570 (PMC4840211; doi:10.3389/fmicb.2016.00570)
Supplement: Supplementary file 1 [file Data_Sheet_1.DOCX]

**Supplementary Tab S1 Transcriptional regulators and their interactions in *A. fumigatus*** obtained by text mining in about 8,000 scientific articles

| TFs | # targets | Other Regulators | # targets |
| --- | --- | --- | --- |
| AbaA | 1 | Cwh41 | 1 |
| AcuM | 2 | FarA | 3 |
| BrlA | 11 | FmqA | 3 |
| GipA | 2 | FmqB | 3 |
| HapX | 4 | FmqC | 2 |
| LaeA | 21 | GprD | 1 |
| PacC | 4 | HdaA | 2 |
| RsmA | 3 | Hsp90 | 4 |
| SebA | 4 | MirB | 1 |
| SrbA | 27 | MtfA | 1 |
| SrbB | 5 | PrtT | 4 |
| SreA | 22 | SidA | 4 |
| WetA | 1 | SidC | 2 |
| Yap1 | 9 | VeA | 3 |
| ZafA | 3 | VosA | 2 |
|  |  | Wsc1 | 1 |


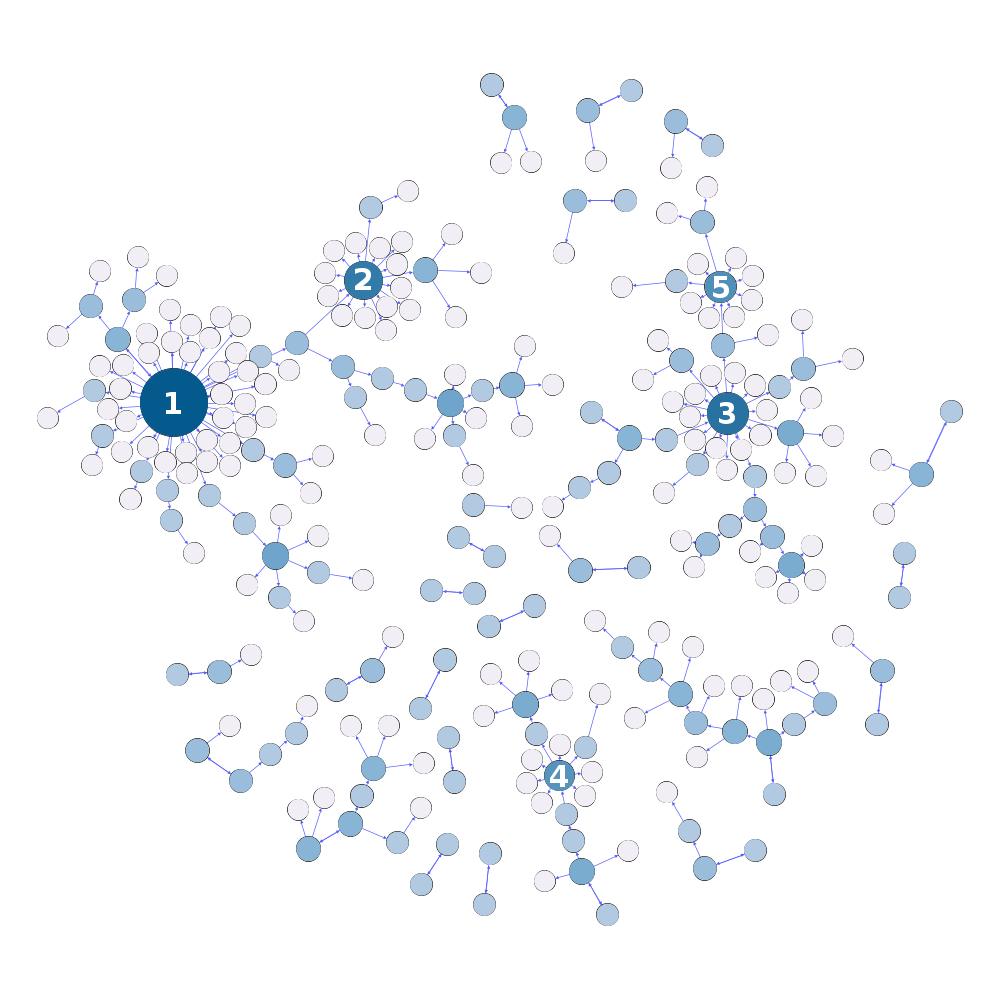


**Supplementary Fig. S1 Inferred GRN network for *A. fumigatus.*** The network is based on 270 filtered genes of the ‘gold standard’. The colour of the nodes depends on the respective outdegree. Hubs: 1 – XlnR (xylanolytic transcriptional activator), 2 – LaeA (regulator of secondary metabolism), 3 – SreA (siderophore TF), 4 – HLH (putative TF), 5 – Atf21 (putative BZIP TF, atfB/atfC)
